# Enhanced radiation sensitivity and radiation recall dermatitis (RRD) after hypericin therapy – case report and review of literature

**DOI:** 10.1186/1748-717X-1-32

**Published:** 2006-09-01

**Authors:** Kurt Putnik, Peter Stadler, Christof Schäfer, Oliver Koelbl

**Affiliations:** 1 Department of Radiation Oncology, University of Regensburg, Josef-Strauss Allee 11, 93053 Regensburg, Germany

## Abstract

**Background:**

Modern radiotherapy (RT) reduces the side effects at organ at risk. However, skin toxicity is still a major problem in many entities, especially head and neck cancer. Some substances like chemotherapy provide a risk of increased side effects or can induce a "recall phenomenon" imitating acute RT-reactions months after RT. Moreover, some phototoxic drugs seem to enhance side effects of radiotherapy while others do not. We report a case of "radiation recall dermatitis" (RRD) one year after RT as a result of taking hypericin (St. John's wort).

**Case report:**

A 65 year old man with completely resected squamous cell carcinoma of the epiglottis received an adjuvant locoregional RT up to a dose of 64.8 Gy. The patient took hypericin during and months after RT without informing the physician. During radiotherapy the patient developed unusual intensive skin reactions. Five months after RT the skin was completely bland at the first follow up. However, half a year later the patient presented erythema, but only within the area of previously irradiated skin. After local application of a steroid cream the symptoms diminished but returned after the end of steroid therapy. The anamnesis disclosed that the patient took hypericin because of depressive mood. We recommended to discontinue hypericin and the symptoms disappeared afterward.

**Conclusion:**

Several drugs are able to enhance skin toxicity of RT. Furthermore, the effect of RRD is well known especially for chemotherapy agents such as taxans. However, the underlying mechanisms are not known in detail so far. Moreover, it is unknown whether photosensitising drugs can also be considered to increase radiation sensitivity and whether a recall phenomenon is possible. The first report of a hypericin induced RRD and review of the literature are presented. In clinical practise many interactions between drugs and radiotherapy were not noticed and if registered not published. We recommend to ask especially for complementary or alternative drugs because patients tend to conceal such medication as harmless.

## Findings

Although the introduction of higher voltage radiotherapy reduced severe cutaneous side effects in the past, today particularly chemotherapy can sensitise skin to radiation resulting an acute skin reactions of higher degree [1-4]. The cutaneous side effect ranges from erythema up to moist epitheliolysis. The wound healing of radiation induced acute side effects is normally finished after some weeks. In literature a phenomenon is described occurring weeks or months after RT and corresponding to the acute skin reaction. This phenomenon is called radiation recall dermatitis (RRD) and may be induced by drugs, however, disappears after removing the inducing substance again. RRD is described for several chemotherapy agents [5]. So far, both a sensitising effect and RRD of drugs apart from chemotherapy are not systematically analysed.

We report on a patient having developed RRD one year after radiotherapy induced by a hypericin (St. John's wort). Additionally a literature overview on photo- and radiation sensitising substances is presented.

## Case report

**Figure 1:**
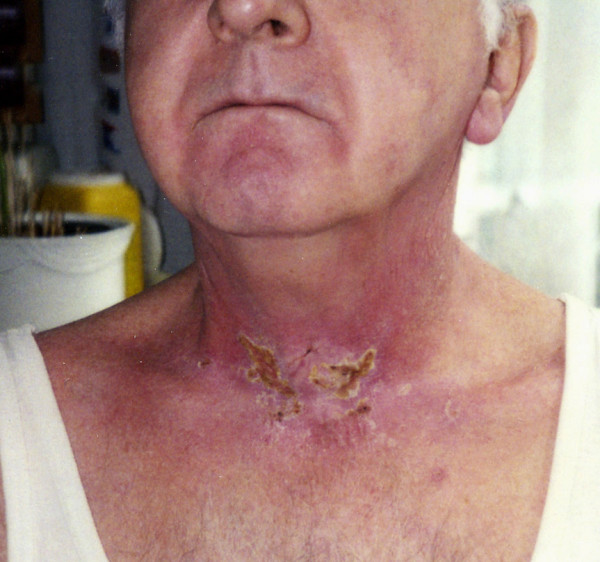
Skin toxicity at the end of radiotherapy (RT). During the forth weeks of the RT the described patient developed a distinctive erythema (WHO II), which changed to moist epitheliolysis (WHO III) at the fifth week. At the end of the radiotherapy moist epitheliolysis with crust occurred. As well, the skin remained hyper-pigmented.

**Figure 2:**
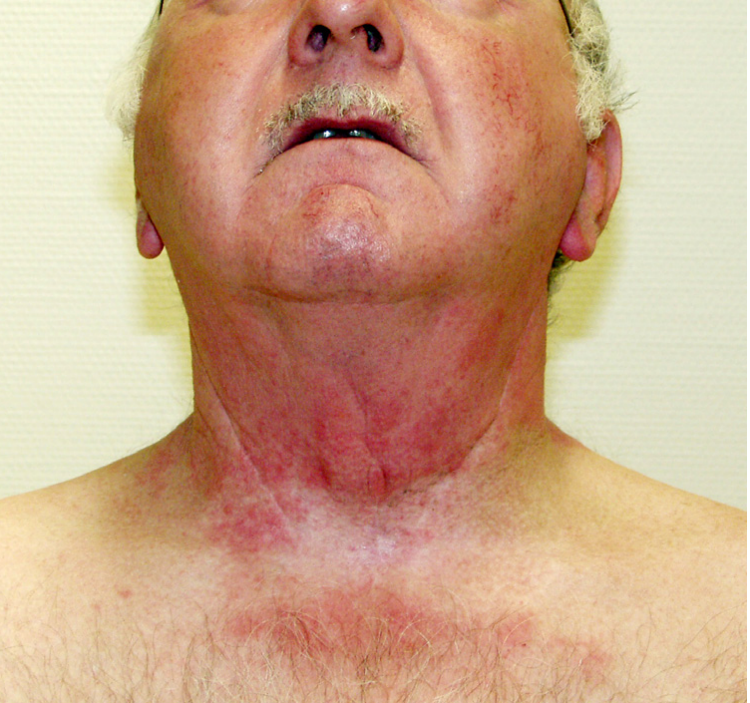
Skin efflorescence after sunbathe one year after RT-end. About a half year after RT in the aftercare the skin was completely recovered, only hyper- and hypopigmentation were visibly. At the regular following date one year after radiotherapy the patient showed a renewed distinctive erythema exclusively within the former irradiated skin region. The erythema appeared after sunbathe and resembled the clinical picture of the previous radiogenic acute-reaction.

**Figure 3:**
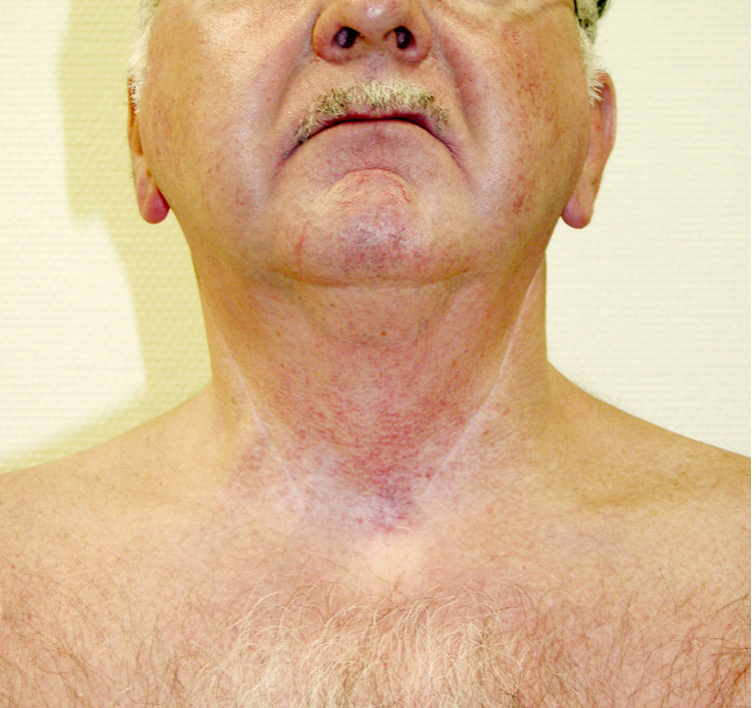
Skin efflorescence after leaving out St. John's wort. Despite the prescription of a steroid containing cream the skin efflorescences recovered, but appeared again unchanged after leaving out the cream for a short time. On a specific questioning the patient reported for the first time to take hypericin (Johanniskraut Sandos 425) within the last few years. After stopping taking the medicine the erythema faded away completely in short time.

A 65-year old patient with a squamous cell carcinoma of the epiglottis diagnosed 11/2003 received a laser-surgically organ preserving operation. From February to April 2004 a postoperative radiotherapy was done encompassing the region of the primary cancer including the cervical and supraclavicular lymphatic regions. Total dose was 64,8 Gy, single dose 1,8 Gy. A multiple-field technique was used by combination of photons and electrons. During the forth week the patient developed a distinctive erythema (WHO II), which changed to moist epitheliolysis (WHO III) at fifth week. At the end of radiotherapy moist epitheliolysis with crust occurred (Figure 1). Five months after radiotherapy the skin was completely recovered, only hyper- and hypopigmentation were visibly. At the regular following date one year after radiotherapy the patient showed a renewed distinctive erythema exclusively within the former irradiated skin region (Figure 2). The erythema rised after sunbathe and resembled the clinical picture of a radiogenic acute-reaction. According to prescription of a cream containing steroids skin-efflorescences recovered, but appeared again unchangedly after going of the cream for a short time. On a specific questioning the patient reported for the first time to take hypericin (Johanniskraut Sandos 425) within the last few years. After stopping taking the medicine the erythema faded away completely in short time (Figure 3).

## Discussion

Different medicinal drugs can sensitise the skin to UV radiation or visible light (photosensitivity), x-rays (radiation sensitivity) or can induce a radiation recall dermatitis (RRD) (Tab. 1) [6]

**Table 1:** Drugs associated with radiation sensitivity, RRD and photosensitivity [6].

**Increased radiation sensitivity and RRD**		
Adriamycin	Etoposid	Methotrexat
Bleomycin	5-Fluorouracil	Tamoxifen
Dactinomycin	Gemcitabine	Tuberculostatic
Daunorubicin	Hydroxyurea	Rimetrexate
Docetaxel	Melphalan	Vinblastin
Doxorubicin	Paclitaxel	

**Photosensitivity**		

Amitriptylin	Felodipin	Norfloxacin
Benzydamin	Indometacin	Sulfadiazin-Silber
Dacarbazin	Meloxicam	Sulfamethoxazol
Diclofenac	Methotrexat	Temoporfin
Doxycyclin	Metoprololsuccinat	Triamcinolonacetonid
5-Fluorouracil	Mitomycin	Trimethoprim
Flurovoxaminmaleat	Natriumaurothiomaleat	Vinblastin

Photosensitivity occurs both as a phototoxic, non-immunologic phenomenon and as a photoallergic, immune-dependent reaction. The much more common phototoxicity can be subdivided into a photodynamic type, which requires oxygen, and a nonphotodynamic, which does not [7]. The majority of photosensitising drugs have an action spectrum within UVA. The photosensitising effect of several substances, e.g. Hypericin, is used for photodynamic therapy against cancer [8,9].

Especially chemotherapeutic agents can increase the radiation sensitivity of the skin. These substances may lead not only to enhanced acute cutaneous side effects, but induce skin efflorescences for months afterwards, which resemble closely the acute skin reaction to radiotherapy. This delayed reaction is called radiation recall dermatitis (RRD), an inflammatory skin reaction after administration of certain promoting agents, such as antineoplastic drugs, in a previously irradiated area. First reports on RRD date back to 60 years. So far there is no systematic overview on incidence and aetiology of RRD but only case reports. In these reports RRD was described as varying from moderately rare to moderately common side effect caused by unknown mechanisms [10]. However, some drugs, especially chemotherapy agents, seem to induce RRD more frequently. Camidge described an increased RRD risk for several chemotherapies, for examples Actinomycin D, Adriamycin or Paclitaxel [10]. Additionally, reports on Tamoxifen and tuberculostatic therapy are found in literature.

The time between the end of radiation and RRD varies extremely. A median of 39 days with a range between 7 and 840 days is reported [10]. The interval between drug and the first appearance of skin reaction depends on the administration. While first skin reaction is described immediately after an intravenous application, it takes some days after oral application. So far, there are no recommendations on a standard therapy of the RRD. On contrary the treatment of the RRD is discussed controversially in literature. Some authors recommend the systemic or local application of steroids or antihistaminics, others refuse [11,12]. The application of steroids may reduce skin reaction, but stopping the therapy may result in a rebound phenomenon as shown in our patient. As such, the role for systemic steroids, topical steroids or anti-histamines in the treatment of acute RRD remains unclear. There is consent that the RRD triggering substance should be removed immediately [10]. In our case report the discontinuation of the drug led to a complete healing of the skin efflorescences in a short time.

The aetiology of the RRD is still unclear. A lot of different hypotheses have been discussed for RRD although there is a little evidence basing to support any of them. The hypotheses include vascular damage, epithelial stem cells inadequacy, epithelial stem cell sensitivity or drug hypersensitivity reactions. Even skin biopsies showing non-specific changes couldn't clear the aetiology of the RRD [11].

Reports on an increased radiation sensitivity induced by non-chemotherapeutic substances are rare in literature. However, there are a lot of drugs with a described photosensitising effect. Photosensitising as a typical side effect is indicated for 76 drugs approved in Germany and listed in " Rote Liste" [13].

Phytotherapy can also induce photosensitising. Phytopharmaca are drugs which contain exclusively plants, plant-parts or plant-components or combinations of it in finished or untreated condition as active components and belong to comparative or alternative medicine. Complementary and alternative medicine is more common among patients with cancer than in the general population [14]. In the 1990s metaanalyses of 26 studies conducted worldwide showed that phytopharmaca were widely used by cancer patients with a prevalence ranging from 75 to 64% [15]. In 2002 a more recent study reported on an increase of this use up to 83% [16]. Therefore the market for alternative drugs has a volume of over 4 billion dollars in the USA and 6.7 billion dollars in Europe [17]. In Germany the total 2003 retail sales were 939 million euros [18].

In the USA up to 72% of the patients with cancer using alternative medicine do not inform their treating physician [19,20]. An estimated 15 million adults combine alternative remedies with prescription medicine [21].

In a recent study Rieger et al. reported that at least 20 % of the hospitalises patients take additional substances without informing the attending physicians. He reported that urine samples of 20% of patients were positive tested for a compound of unknown co-medication [22]. Martin-Facklam et al. evaluated the extent of systemic exposure to St. John's wort in patients on admission and during hospital stay, and compared the results with known use of St. John's wort as documented in the drug chart and detected in additional interviews. Hyperforin or hypericin were detected in 11.3 % of patients. Six percent of patients had taken St. John's wort without the knowledge of the medical team and the pharmacist, in spite of additional interviews and seven of these patients were treated concurrently with drugs that can interact with St. John's wort [23]. In the USA more than 100000 deaths per year can be attributed to drug interaction and it has been suggested that the greater part of these might be linked to the use of herbs [24,25].

St. John's wort is one of the most extensively studied Hypericum. Today St. John's wort is widely used for the treatment of mild to moderate depression and other nervous conditions [15,26]. It is a complex mixture of more than two dozen compounds influencing drug-metabolising enzymes, drug transporters and pharmacokinetic [27], e.g. relevant for patients using Digoxin [28], Theophyllin [29], Cyclosporine [30], oral Contraceptive [31], Phenprocoumon [32], Warfarin [33] and Sertaline [34]. Additionally a photo sensitising effect of St. John's wort is well described in the literature of human as well as veterinary medicine [8,9,35-37]. Beattie et al. describe decreased erythemal threshold to ultraviolet A1 irradiation as mechanism for the photo sensitising effect of St John's wort [38]. Zhang et al. even reported on an enhancement of radiation sensitivity by Hypericin in glioma cells [39].

In patients, who self-prescribe herbal medicinal products, the risk of increased cutaneous side effects by radiotherapy like the reported RRD is enhanced. So fare the mechanism responsible for the increased radiation sensitivity is unclear. As described above, herbal medicines are used by a major part of patients with cancer, which are irradiated frequently in combination with chemotherapy.

Retrospectively it may be suggested that the acute side effects during radiotherapy were enhanced by the additional use of St. John's wort in our reported case. But it may safely assert that the RRD one year after radiotherapy was induced by St. John's wort, because RRD healed up by discontinuity of its use.

Phytopharmaca are frequently used by a major part of patients with cancer. Therefore the radiation oncologist regularly sees patients who self-describe herbal medicinal products but do not volunteer this information. This co-medication can increase the toxicity of anticancer therapy. In the reported case the acute and the late cutaneous toxicity was increased probably by a radiation sensitising effect of St. John's wort. As a consequence of this all patients should be questioned about co-medicine, especially complementary and alternative medicine like phytopharmaca.

## Competing interests

The author(s) declare that they have no competing interests.

## Authors' contributions

KP reviewed patient data and drafted the manuscript. PS and CS participated in the collection and analysis of the data. OK participated in the conception of manuscript as well as the interpretation of the data of literature and drafting the manuscript. All authors read and approved the final manuscript.
